# Adjuvant Therapy in Adrenocortical Carcinoma: Reflections and Future Directions

**DOI:** 10.3390/cancers12020508

**Published:** 2020-02-22

**Authors:** Sara Bedrose, Marilyne Daher, Lina Altameemi, Mouhammed Amir Habra

**Affiliations:** 1 Department of Endocrine Neoplasia and Hormonal Disorders, Unit 1461, The University of Texas MD Anderson Cancer Center, 1515 Holcombe Boulevard, Houston, TX 77030, USA; ssbedrose@mdanderson.org (S.B.); mdaher1@mdanderson.org (M.D.); LAltameemi@mdanderson.org (L.A.); 2 Department of Endocrinology, Diabetes and Metabolism, Baylor College of Medicine, Houston, TX 77030, USA

**Keywords:** adrenocortical carcinoma, survival, recurrence, chemotherapy, mitotane, radiation therapy

## Abstract

Adrenocortical carcinoma (ACC) is a rare and aggressive malignancy with high risk of recurrence despite macroscopically complete surgical resection. The main predictors of ACC recurrence include advanced disease stage, incomplete surgical resection, cortisol production, certain genetic alterations, and high proliferation rate (Ki-67 proliferation index). Mitotane has been the mainstay adjuvant therapy of ACC. However, the use of mitotane is based on retrospective and occasionally conflicting evidence. As mitotane levels can take a few months before reaching therapeutic levels, there is an emerging practice of combining platinum-based chemotherapy with mitotane in the adjuvant setting. Retrospective data indicate that radiotherapy is an option for select patients, particularly those with positive resection margins. There are multiple knowledge gaps in selecting patients for adjuvant therapy. It is of great importance to establish risk calculators to predict recurrence and to implement molecular profiling of ACC to guide adjuvant therapy. The role of immunotherapy in metastatic ACC is emerging and if deemed efficacious, then future studies will be needed to ascertain the role of adjuvant immunotherapy in ACC.

## 1. Introduction

Adrenocortical carcinoma (ACC) is an orphan malignancy that affects 1–2 people per million each year [1,2,3]. Most reports about adjuvant therapy in ACC come from case series and retrospective data analyses, mostly originating from tertiary referral centers [4,5,6,7].

Since the first ACC case series was described by Otto Ramsay in 1899 [8], many milestones have been achieved in the disease’s treatment and management, including the introduction of cortisone in the 1940s [9] and mitotane in the 1960s [10]. Recent studies indicate that a higher percentage of patients are diagnosed in earlier stages (II–III) [7,11]. This is in contrast to earlier studies, in which most patients presented with stage IV disease [12]. This difference may be due to improved imaging technology that has led to earlier diagnosis in some patients before becoming symptomatic. Complete surgical resection is the mainstay of treatment of localized disease, although the recurrence rate can be as high as 70% according to some studies [13]. Adjuvant therapy to reduce the risk of ACC recurrence was reported almost five decades ago [4,14]. Since then, many studies have taken place in an attempt to guide the optimal use of adjuvant therapy for ACC. In this manuscript, we summarize key data to support the use of adjuvant treatment to improve the clinical outcomes of patients with ACC. We also discuss knowledge gaps and provide suggestions for future studies and tools to help treat ACC patients after surgery. 

## 2. Risk of Recurrence of ACC Compared with Other Cancers 

Adjuvant therapy reduces the risk of recurrence in many cancers, although its use in ACC is still a matter of debate. For example, the estimated risk of recurrence in patients with triple-negative breast cancer is approximately 43%, and 25% in patients with non-triple-negative breast cancer [15]. A meta-analysis performed by Early Breast Cancer Trialists’ Collaborative Group found that patients with breast cancer treated with adjuvant anthracycline-based regimen (8575 patients) had a 10 year recurrence rate of 39.4% compared to 47.4% in those who did not receive any adjuvant chemotherapy (risk ratio [RR], 0.73; 95% confidence interval [CI], 0.68–0.79), translating into a number needed to treat (NNT) of 13 (i.e., one recurrence is prevented for each 13 patients treated). The same study found a 5% reduction in 10 year overall mortality among the patients receiving adjuvant anthracycline-based regimen (RR, 0.84; 95% CI, 0.78–0.91), translating to a NNT of 20 to add one survivor at 10 years [16]. 

In patients with colon cancer, the recurrence rate among patients who present with stage III disease is estimated to be approximately 40%, and approximately 12% in those presenting with stage II disease [17]. The benefits of fluoropyrimidine-based adjuvant systemic therapy have been shown primarily in stage III colorectal cancer. A study in mucinous colon adenocarcinoma patients showed a significant difference in overall survival (OS) between patients with stage II disease who received adjuvant chemotherapy and those who did not (hazard ratio [HR], 0.79; 95% CI, 0.69–0.90). This benefit was even greater for patients with stage III disease (HR, 0.56; 95% CI, 0.52–0.60) [18]. 

In patients with non-small-cell lung cancer, the recurrence rate is estimated to be approximately 40% [19]. A systematic review of 8447 patients who had undergone surgery for early stage non-small-cell lung cancer found that adjuvant chemotherapy conferred a 5 year OS of 64% compared to 60% in patients who did not undergo adjuvant therapy (HR, 0.86; 95% CI, 0.81 to 0.92) [20], making the NNT to add one survivor at 5 years to be 25. In another meta-analysis of 2660 patients, adjuvant chemotherapy conferred an advantage when added to surgery and radiotherapy in patients with early non-small-cell lung cancer, with a 5 year survival rate of 33% compared with 29% in those who had only surgery and radiotherapy (HR, 0.88; 95% CI, 0.81 to 0.97), making the NNT approximately 25 patients to add one survivor at 5 years. 

In ACC, even after complete surgical resection, the estimated recurrence rate of ACC is 40% to 70% [13], which is higher than other common malignancies. Thus, until prospective data become available, the use of adjuvant therapy in ACC can be justified based on biological and clinical backgrounds.

### 2.1. Predictors of Recurrence in ACC

#### 2.1.1. Stage

Disease stage is an important prognostic predictor of ACC recurrence. A study of 416 patients from the German ACC Registry showed disease-specific survival to be stage-dependent. The 5 year survival rates for patients with stage I, II, III, and IV disease were 82%, 58%, 55%, and 13%, respectively [21]. Another study on 275 patients found that patients with local disease (defined in the study as stages I–II) had longer recurrence-free survival (RFS) rates compared to those with regional (defined as stage III) and distant (defined as stage IV) disease, after undergoing surgical resection (*p* = 0.026). Multivariate analysis in this study also showed that patients with local disease had longer OS rates than those with stage III/IV disease (HR, 0.438; 95% CI, 0.325–0.590) [22].

#### 2.1.2. Hormonal Status

Several studies have suggested that cortisol-producing ACCs are associated with worse prognosis. A study of 202 patients with varying disease stages found that cortisol hypersecretion was an independent risk factor for worse prognosis in ACC (HR, 3.94; 95% CI, 2.22 to 6.99) [6]. Another retrospective study of 72 ACC patients showed similar results wherein cortisol hypersecretion, regardless of androgen hypersecretion, was associated with shorter OS (HR, 0.641; 95% CI, 0.422–0.973) [23]. A multicenter, multinational study of 524 patients with completely resected, non-metastatic ACC showed that cortisol overproduction was associated with shorter RFS (HR, 1.30; 95% CI, 1.04–2.62) and OS (HR, 1.55; 95% CI, 1.15–2.09), after adjusting for other factors [24]. This large study did not find a significant association between cortisol overproduction and mitotic index (*p* = 0.56), an indicator of tumor proliferation activity. Thus, the exact mechanism by which cortisol hypersecretion is associated with worse outcomes in ACC patients remains unclear but could be related to suppression of immune surveillance of ACC.

#### 2.1.3. Resection Margin 

Positive surgical resection margin is associated with worse outcomes in many cancers, including hepatocellular and colorectal carcinomas [25,26]. Multiple studies have found that complete surgical resection (R0) of ACC is associated with better outcomes and longer RFS and OS [7,22,27,28]. In fact, preliminary data from one study suggested that patients with stage III disease with complete surgical resection (R0) had better survival outcomes than those with stage II disease who had histologically incomplete resection [21]. A meta-analysis of more than 1500 patients identified from the National Cancer Database found that patients with R0 margins had a median OS of 57.6 months (95% CI, 48.5–66.0), which was significantly longer than that of patients with microscopically positive (R1) margins (22.4 months; 95% CI, 17.6–33.5), and that of patients with macroscopically positive (R2) margins (13.7 months; 95% CI, 5.8–26.8) [29]. The 5 year OS rates for patients with R0, R1, and R2 resection status were 48.8% (95% CI, 45.7%–51.8%), 28.6% (95% CI, 21.0%–36.8%), and 22.6% (95% CI, 9.4%–39.2%), respectively. In contrast to these findings, a multi-institutional study assessing the relationship between resection status and RFS did not find resection status to be an independent risk factor for shorter RFS (HR, 1.06; 95% CI, 0.58–1.94). However, it was noted to be an independent predictor of worse OS (HR, 2.22; 95% CI, 1.03–4.77) [30]. The different findings of these studies could be attributed to variations in the volume of cases and clinical experience in ACC management between institutions. National studies are more likely to have larger variations in experience while studies from high volume referral centers are more reflective of higher experience and multidisciplinary approach in ACC management that has been linked to better outcomes [31].

#### 2.1.4. Type of Surgery: Laparoscopic vs. Open Adrenalectomy

Treatment guidelines recommend open surgery via transperitoneal access as the standard treatment for suspected ACC. Laparoscopic adrenalectomy is safe to perform on ACCs with certain conditions, including small tumor size (<6 cm) and no evidence of tumor invasiveness, if performed in centers with significant experience in laparoscopic adrenal surgery [32]. A study assessing surgical margins of 2117 patients identified through the National Cancer Database found that 19.4% of patients who underwent surgical resection had positive margins—9% of whom had R1 margins and 10% of whom had R2 margins [33]. In another analysis, laparoscopic resection was associated with higher recurrence rates (*p* < 0.0001) but shorter operative time, less estimated blood loss, and shorter postoperative hospital stay compared to open approach [34]. 

#### 2.1.5. Ki-67

The Ki-67 labeling index is a very important factor in predicting ACC recurrence following R0 resection. In a retrospective study that included 17 ACC patients, a Ki-67/MIB-1 labeling index of 7% or higher was associated with shorter disease-free survival durations (*p* = 0.0037) [35]. Another study, which compared 319 patients from the German ACC Registry with 250 patients in a validation cohort found Ki-67 to be the single best prognostic value for RFS (HR for recurrence, 1.042 per 1% increase; *p* < 0.0001) and OS (HR for death, 1.051; *p* < 0.0001) [36]. Clinical outcomes also varied significantly according to patients’ Ki-67 index. Patients with Ki-67 <10%, 10%–19%, and ≥20% had median RFS of 53.2, 31.6, and 9.4 months, respectively, and median OS of 180.5, 113.5, and 42.0 months, respectively. Ki-67 also proved to be superior to other histological scores. It was used to establish a grading system for ACC with grade 1, 2, and 3 tumors defined as Ki-67 of <10%, 10%–19%, and ≥20%, respectively [36]. Thus, reporting Ki-67 percentage is an essential component in pathology reports of patients with ACC [36].

#### 2.1.6. Molecular Profile

ACC is associated with multiple hereditary cancer syndromes including Li-Fraumeni Syndrome, Beckwith–Wiedemann Syndrome, Carney Complex, multiple endocrine neoplasia type I [37,38]. A recent study showed that adrenal cancer was detected in 3.2% of patients with Lynch Syndrome [39]. Interestingly, adrenal cancer was even found to be the presenting cancer is some patients with this syndrome. These findings as well as multiple other retrospective studies demonstrated the prognostic value of molecular classification of ACC. The use of genetic markers as a prognostic factors in ACC has been postulated with data available since 2003 [40]. In 2016, a comprehensive genomic characterization of 91 cases of ACC was done as part of the Rare Cancer Project of The Cancer Genome Atlas [41]. The study, which included tumor samples from patients from four continents to provide a nearly worldwide perspective, confirmed several alterations to be critical for ACC tumorigenesis and progression. The main mutated genes found in ACC included *TP53*, *ZNFR3*, *CTNNB1*, *PRKAR1A*, *CCNE1*, and *TERF2* [41], and whole-genome doubling was identified as a hallmark of ACC progression. The study integrated the ACC subsets identified through a Cluster of Cluster (CoC) analysis with three resulting subtypes with distinct clinical outcomes and molecular alterations [41]. The rates of disease progression were 7%, 56%, and 96% for CoC subtypes I, II, and III, respectively. Median event-free survival was only 8 months for patients with CoC III ACC, and was not reached for those with CoC I. Outcomes varied for patients with CoC II, who had a median event-free survival of 38 months. Stage III/IV tumors accounted for 25%, 47%, and 52% of CoC I, II, and III ACCs, respectively; and stage I/II tumors classified as CoC III had Ki-67 index scores that corresponded with their grade classification [41]. Another study revealed that VAV2 overexpression induced by increased steroidogenic factor-1 is necessary for ACC tumor cell invasion [42]. VAV2 expression and Ki-67 index were significantly correlated with each other as well as with progression-free and OS. Furthermore, patients could be stratified into low- or high-risk groups according to their VAV2 expression and Ki-67 level [42]. Another study found that the *TOP2A*, *NDC80*, *CEP55*, *CDKN3*, and *CDK1* genes were overexpressed in ACC compared with normal adrenocortical tissues [43]. Most recently, a multicenter retrospective biomarker analysis included 368 patients who had undergone resection of localized ACC. For patients with stage I–III disease, molecular classification was an independent prognostic factor for disease-free survival and the combination of tumor stage, tumor grade, and molecular classification offered the best predictive model for disease-free survival [44]. Another approach recently divided prognostic factors into three groups: clinical factors (age, stage, and hormonal symptoms), pathological factors (Weiss Score, mitotic count, SF-1, Ki-67, AVA2, P53, beta-catenin, resection margin status), and molecular factors (methylation profile, chromosomal aberrations, and miRNA expression, gene mutations) [45]. Based on these data, combining clinical, pathological, and molecular factors was the best way to stratify ACC.

### 2.2. Prognostic Calculators 

Several prognostic risk scores and calculators have been proposed to help with postoperative management of ACC. However, none of these scores and calculators have been prospectively validated or used to select patients for adjuvant therapy. The first such system, the Helsinki score, combined morphological (mitoses and necrosis) and immunohistochemical parameters. This score includes 3 × mitotic rate + 5 × presence of necrosis + proliferation index. The Helsenki system was associated with 100% sensitivity and 99.4% specificity to diagnose metastatic ACC compared to a sensitivity of 100% and specificity of 90.2% with Weiss score [46]. Using a composite prognostic score (Ki-67 index, tumor size, and the presence of venous tumor thrombus) can be also used to predict ACC outcome but not validated to select patients for adjuvant therapy [36]. More recently, grade, R status, age, and symptoms (GRAS) score was established through a retrospective study of 65 patients who had undergone adrenalectomy for ENSAT stages I–III ACC from 2009 to 2017 [47]. Higher GRAS scores were associated with worse OS (HR, 2.7; 95% CI, 1.43–5.11) and RFS (HR, 3.31; 95% CI, 1.68–6.52).

## 3. Adjuvant Therapy for ACC

### 3.1. Adjuvant Mitotane 

Mitotane is a unique antineoplastic agent used solely in the treatment of ACC. It is an isomer of dichloro-diphenyl-trichloroethane (DDT) that is directly cytotoxic to adrenal tissue. Several mechanisms have been postulated for its action including alteration of mitochondrial respiratory chain activity by inducing cytochrome c oxidase defect in adrenocortical cells [48]. The active form of mitotane irreversibly binds with adrenal molecules, including cytochrome P450 (CYP) enzymes such as CYP11A1, CYP11B1, and CYP21B, causing altered concentrations of hormones in the serum [49]. Mitotane directly interacts with lipid membranes and inhibits sterol-O-acyl-transferase activity, resulting in an excess of free cholesterol and other fatty acids that are toxic to hormone-producing adrenal cells [50,51]. Mitotane affects the proteins involved in stress response, tumorigenesis, and cellular metabolism and structure [52]. As mitotane inhibits steroidogenesis by multiple mechanisms, it also induces CYP3A4 expression, leading to increased steroid clearance; thus, patients who receive mitotane often require higher steroid doses than usual. Mitotane’s pharmacokinetic properties are not fully elucidated. It has several drug interactions through induction of metabolizing enzymes.

Treatment with mitotane can be challenging in some patients. Side effects of mitotane therapy are common and can include anorexia, nausea and vomiting, among others. Potentially severe adverse events include adrenal insufficiency and crisis [53].

Although mitotane is approved in metastatic ACC—mitotane efficacy requires reaching a target therapeutic level between 14 and 20 mg/L—its use in the adjuvant setting is still controversial and lacks predictors of response. There is also uncertainty about the target level needed to prevent recurrence in this setting [54].

An early retrospective study of adjuvant mitotane therapy for ACC included patients treated from 1950 to 1972 [14]. Of the nine patients who received adjuvant mitotane after surgery, five survived three years, and four survived five years or longer. However, there was no statistical difference in OS between patients treated with surgery and adjuvant mitotane and those treated with surgery alone. Another small study included seven patients who received adjuvant mitotane between 1950 and 1981—of whom, six were alive at 1 to 4 years [55]. In 1993, a study on 19 patients who had localized or regional disease at the time of surgery found no benefit in survival or disease-free interval in patients who received adjuvant mitotane compared with those treated with surgery alone [56]. Additionally, adjuvant therapy did not improve disease-free interval and survival. The support for adjuvant mitotane use came from a retrospective study of 177 patients with ACC who had undergone radical surgery at 8 centers in Italy and 47 centers in Germany between 1985 and 2005 [57]. The adjuvant mitotane group included 47 Italian patients, control group 1 included 55 Italian patients, and control group 2 included 75 German patients. Median RFS was significantly longer in the mitotane group (42 months), compared with 10 months in control group 1 (HR, 2.91; 95% CI, 1.77–4.78), and 25 months in control group 2 (HR, 1.97 (95% CI, 1.21–3.20) [57]. A subsequent retrospective single institution study of 218 patients who underwent primary resection of ACC showed that disease-free survival was better with adjuvant mitotane, although it did not reach statistical significance (HR, 1.76; 95% CI, 0.98–3.16). No statistically significant difference was seen in OS between the two groups (HR, 1.41; 95% CI, 0.69–2.88) [58]. A systematic review and meta-analysis in 2018 including 5 retrospective studies and 1249 patients found that adjuvant mitotane was associated with longer RFS (HR, 0.62; 95% CI, 0.42–0.94) and OS (HR, 0.69; 95% CI, 0.55–0.88). 

Further support for adjuvant mitotane came from another recent retrospective study that included 152 patients with non-metastatic ACC (100 patients who received adjuvant mitotane and 52 who did not receive adjuvant therapy) who were stratified by disease stage (I-II vs III), hormone secretion (yes vs. no), and Ki-67 index. The recurrence risk was greater in patients who did not receive mitotane (HR, 2.79; 95% CI, 1.58–4.91) compared with those who did, but OS did not differ significantly between groups [59]. 

The first prospective study on the use of adjuvant mitotane, ADIUVO trial, aims to compare the use of adjuvant mitotane to observation in ACC patients with low to intermediate risk of recurrence (ClinicalTrials.gov identifier, 777244). Endpoints of this study are disease-free survival (primary endpoint), overall survival, and quality of life.

**Figure 1:**
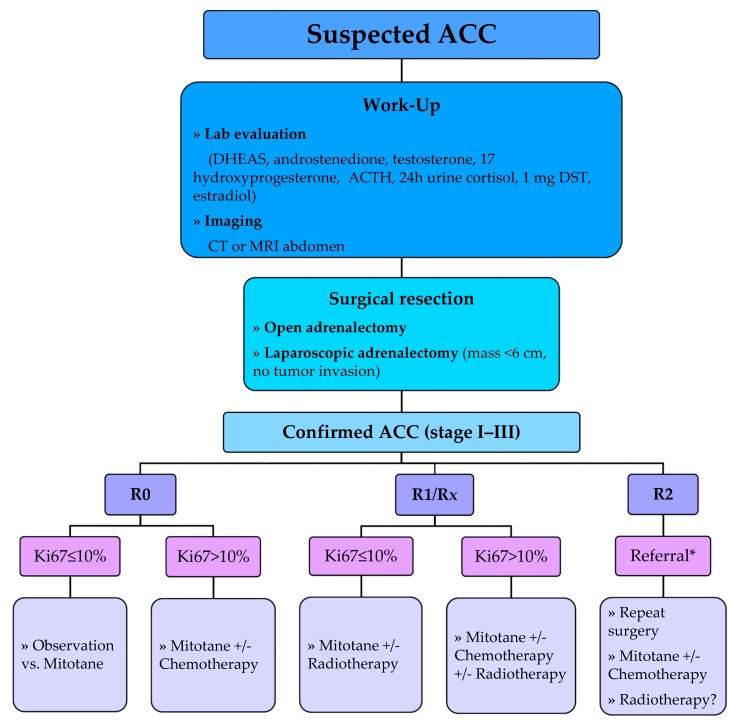
Suggested algorithm of the management of localized adrenocortical carcinoma. DST, dexamethasone suppression test; R0, no evidence of tumor; R1, microscopic evidence of tumor; R2, macroscopic residual disease; RX, margins unknown. * If initial surgery was done outside major referral centers.

The European Society of Endocrinology (ESE) and ENSAT 2018 guidelines recommend adjuvant mitotane treatment following resection of ACC in patients considered to have a high risk of recurrence (i.e., stage III, R1 resection, or Ki-67 > 10%) [32] as demonstrated in Figure 1. In this setting, adjuvant mitotane should be given for at least 2 years but no longer than 5 years. These recommendations are based on data from a meta-analysis of six studies that assessed ACC recurrence (pooled HR, 0.7; 95% CI, 0.5–1.1) and mortality (pooled HR of five studies, 0.7; 95% CI, 0.5–0.9) in patients treated with mitotane. However, the guidelines could not recommend for or against adjuvant mitotane therapy for patients at low or moderate risk of recurrence (i.e., stage I–II, R0 resection, and Ki-67 ≤ 10%) and propose discussing such patients’ adjuvant therapy options on an individual basis. 

### 3.2. Adjuvant Chemotherapy 

The use of cytotoxic chemotherapy alone in the adjuvant setting or in combination with mitotane is still a matter of debate with limited data available. The combination of cisplatin and etoposide was explored in the adjuvant setting in a small series of five ACC patients aged 1 to 21 [60]. All received etoposide (165 mg/m^2^) and cisplatin (90 mg/m^2^) every 3 to 4 weeks for at least six cycles, beginning shortly after surgical resection, and all remained in remission 29 to 109 months later. However, ACC in children has a different natural history than that arising in adults, and there are no data addressing the benefit of adjuvant cisplatin plus etoposide in adults with ACC. The combination of mitotane plus streptozocin as adjuvant therapy was also tested in a phase II trial of 17 patients who had undergone complete tumor resection. Disease-free survival was significantly longer in patients who received combination mitotane-streptozocin adjuvant therapy compared with a separate cohort of 11 patients who received no adjuvant therapy (49 vs. 12 months) [61]. 

In clinical practice, some centers treat high-risk ACC patients (Ki-67 > 10%) with mitotane alone for 2 to 3 years, and other centers administer a combination of mitotane with platinum-based chemotherapy. As mitotane levels usually remain sub-therapeutic in the first few months [62], combining mitotane with chemotherapy in the first 3 months of adjuvant therapy has been used to treat potential micro-metastases until mitotane levels approach therapeutic range. Thus far, there are no published data to validate the superiority of either approach.

In an attempt to identify a standard of care for adjuvant therapy in patients with high-risk ACC, the ADIUVO-2 trial was established. ADIUVO-2 is a randomized registry trial of adjuvant mitotane versus mitotane with cisplatin/etoposide after primary surgical resection of localized ACC with high risk of recurrence (stage I–III, and Ki-67 >10%). The study aims to enroll 240 patients (120 in each arm) and is ongoing (ClinicalTrials.gov identifier, NCT03583710). Endpoints of this study are recurrence-free survival (primary endpoint), overall survival, clinical outcomes, toxicities, and quality of life.

ESE and ENSAT 2018 guidelines did not reach a consensus on the adjuvant use of cytotoxic agents. Although the panel advises against the routine use of cytotoxic agents in the adjuvant setting, it adds that adjuvant chemotherapy should be considered in select patients with ACC with a very high risk for recurrence [32]. 

### 3.3. Adjuvant Radiotherapy 

Retrospective studies on the use of adjuvant radiotherapy in ACC have shown mixed results. In one study, 14 patients receiving adjuvant radiotherapy (XRT) postoperatively were matched with 14 patients who did not receive adjuvant XRT. Patients in the adjuvant group had a lower risk for local recurrence 5 years postoperatively (79% vs. 12%; 95% CI, 53–100%) without a significant difference in distant recurrence or OS (*p* = 0.9) [63]. A retrospective study of 16 patients who underwent adjuvant XRT did not show a significant benefit in local recurrence–free rates at 5 years (*p* = 0.53), distant RFS (*p* = 0.33, in multivariate analysis), and OS (*p* = 0.26 in multivariate analysis) when compared with 32 matched patients who did not undergo adjuvant XRT [64]. A study of 20 matched patients showed that adjuvant XRT conferred a statistically significant benefit in local control (HR, 12.59; 95% CI, 1.62–97.88) without an effect on OS (HR, 1.97; 95% CI, 0.57–6.77) [65]. According to a recent study of 171 patients, adjuvant XRT was more likely to be given to those with positive resection margins [66]. Although no difference in OS was found in the general cohort of patients receiving adjuvant XRT, a 40% reduction in yearly risk of death was found in those with positive resection margins who received adjuvant XRT (HR, 0.60; 95% CI, 0.40–0.92) [66]. In a meta-analysis, adjuvant radiotherapy had a significant benefit on local recurrence (RR, 0.24; 95% CI, 0.12–0.49), but no effect on disease survival and OS [67]. Most recently, a single-institution, propensity-matched retrospective analysis evaluated adjuvant XRT and found that using adjuvant XRT was associated with improved local RFS, all RFS, and OS [68]. So far, there is no prospective data to ascertain the value of adjuvant radiotherapy and findings of retrospective data need to be carefully interpreted because of the potential of referral and selection biases.

The ESE/ENSAT 2018 guidelines did not reach a consensus on the use of adjuvant radiotherapy for ACC [32]. However, the guidelines recommend against the routine use of radiotherapy in patients with stage I–II disease and R0 resection in addition to the consideration of radiotherapy in addition to mitotane therapy on an individual basis in patients with stage III disease or R1 or Rx resection. 

## 4. Future Directions

In the future, it is crucial to have clinically useful prognostic tools to more accurately assess the risk of ACC recurrence in order to guide the use of adjuvant therapy. This may require worldwide multicenter collaborations to reach solid conclusions. For example, prognostic risk calculators have been in use for risk predication in other malignancies [69,70]. Although we have some prognostic calculators for ACC [36,46,47], none have been prospectively validated, which limits their use in decision-making. Thus, there is a need to build a prognostic risk calculator that can be validated prospectively using some or all of the aforementioned prognostic markers. Another useful tool would be using steroid profiling as an early diagnostic as well as prognostic marker. Initial data have been in favor of its use [71,72,73]; however, this tool is not prospectively validated, which limits its use in clinical practice. Based on the data we have regarding genetic markers in patients with ACC [41,42,43,74], using genetic profiling to predict recurrence risk is another important tool. It has been used successfully as a prognostic marker in other malignancies, including breast cancer [75]; however, studies are needed to prospectively validate its use for ACC. Finally, although we have some evidence regarding the potential benefit of immunotherapy in ACC [76,77], there are no data available regarding its use in the adjuvant setting. We are hoping that this option will be explored in select patients in the near future. 

## 5. Conclusions

Adrenocortical carcinoma is a rare and aggressive malignancy with high risk of recurrence. The main predictors of recurrence include advanced disease stage, incomplete surgical resection, cortisol production, certain genetic alterations, and high proliferation rate (Ki-67 proliferation index). There are multiple knowledge gaps in selecting patients for adjuvant therapy. It is crucial to have clinically useful prognostic tools in the future to accurately assess the risk of ACC recurrence in order to guide the use of adjuvant therapy. This includes establishing prognostic risk calculators that are prospectively validated as well as using molecular profiling of ACC. Finally, the role of adjuvant immunotherapy is worth exploring in future studies.
